# TIGIT regulates CD4^+^ T cell immunity against polymicrobial sepsis

**DOI:** 10.3389/fimmu.2024.1290564

**Published:** 2024-03-13

**Authors:** Xuexin Zhong, Haiping Xie, Shuang Wang, Tingting Ren, Junlin Chen, Yuefang Huang, Niansheng Yang

**Affiliations:** ^1^ Department of Rheumatology and Clinical Immunology, the First Affiliated Hospital, Sun Yat-sen University, Guangzhou, China; ^2^ Department of Pediatrics, the First Affiliated Hospital, Sun Yat-sen University, Guangzhou, China

**Keywords:** TIGIT, CD4 + T cells, sepsis, anti-bacterial responses, clp

## Abstract

**Background:**

Sepsis is one of the major causes of death and increased health care burden in modern intensive care units. Immune checkpoints have been prompted to be key modulators of T cell activation, T cell tolerance and T cell exhaustion. This study was designed to investigate the role of the negative immune checkpoint, T cell immunoglobulin and ITIM domain (TIGIT), in the early stage of sepsis.

**Method:**

An experimental murine model of sepsis was developed by cecal ligation and puncture (CLP). TIGIT and CD155 expression in splenocytes at different time points were assessed using flow cytometry. And the phenotypes of TIGIT-deficient (TIGIT^-/-^) and wild-type (WT) mice were evaluated to explore the engagement of TIGIT in the acute phase of sepsis. In addition, the characteristics were also evaluated in the WT septic mice pretreated with anti-TIGIT antibody. TIGIT and CD155 expression in tissues was measured using real-time quantitative PCR and immunofluorescence staining. Proliferation and effector function of splenic immune cells were evaluated by flow cytometry. Clinical severity and tissue injury were scored to evaluate the function of TIGIT on sepsis. Additionally, tissue injury biomarkers in peripheral blood, as well as bacterial load in peritoneal lavage fluid and liver were also measured.

**Results:**

The expression of TIGIT in splenic T cells and NK cells was significantly elevated at 24 hours post CLP.TIGIT and CD155 mRNA levels were upregulated in sepsis-involved organs when mice were challenged with CLP. In CLP-induced sepsis, CD4^+^ T cells from TIGIT^-/-^ mice shown increased proliferation potency and cytokine production when compared with that from WT mice. Meanwhile, innate immune system was mobilized in TIGIT^-/-^ mice as indicated by increased proportion of neutrophils and macrophages with potent effector function. In addition, tissue injury and bacteria burden in the peritoneal cavity and liver was reduced in TIGIT^-/-^ mice with CLP induced sepsis. Similar results were observed in mice treated with anti-TIGIT antibody.

**Conclusion:**

TIGIT modulates CD4^+^ T cell response against polymicrobial sepsis, suggesting that TIGIT could serve as a potential therapeutic target for sepsis.

## Introduction

Sepsis is a life-threatening organ dysfunction resulting from dysregulated response to infection (1). Worldwide, it is estimated that 11 million people die from the disease each year (2). Although there has been a global improvement in clinical outcomes due to improved treatment practices over the preceding decades, mortality rates remain unacceptably high, ranging from 25% to 30% for sepsis and 40% to 50% for septic shock (3, 4). In addition, many sepsis survivors have long-term physical and cognitive impairments, burdening society and the health care system (5).

Sepsis is characterized by disrupted balance between pro-inflammatory and anti-inflammatory responses (6). During sepsis, both innate and adaptive immune system are activated to fight against bacteria, with T cells playing a crucial role (7). Upon bacterial challenge, T cell could activate, proliferate, differentiate and secret various chemokines and pro-inflammatory cytokines to assist microbial pathogens elimination (8–10). In addition, IFN-γ derived from T helper cells 1 (Th1) could augment macrophages antimicrobial effectiveness by increasing microbial phagocytosis, improved antigen presentation ability and upregulated costimulatory molecules for T cell activation (11). Meanwhile, regulatory T cells (Tregs) are induced to control excessive inflammatory responses and cytokine storm, which is related to the severity and mortality on the initial phase (12). It is now recognized that sepsis is also associated with profound and sustained immunosuppression, which on the contrary could lead to T cell death, proliferation and function impairment, upregulation of co-inhibitory molecules, and differentiation inhibition (13). However, the exact regulatory molecule for T cell behavior during sepsis is unclear.

T cell activation needs specific recognition of cognate antigenic peptides presented by MHC molecules and costimulatory signal transmitted by CD28-B7 superfamily molecules interaction (14). Upon activation, coinhibitory receptors, also known as immune checkpoints, are upregulated on the surface of effector T cells and Tregs cells to prevent uncontrolled T cell proliferation and responses (15, 16). T cell immunoglobulin and ITIM domain (TIGIT) is a novel immune check point originally identified as a member of the Ig superfamily of molecules, which mainly expressed on activated CD4^+^ T cells, CD8^+^ T cells, NK cells, Tregs and follicular helper T cells (Tfh) (17). The structure of TIGIT contains an extracellular IgV domain, a transmembrane region, and cytoplasmic immunoreceptor tyrosine-based inhibitory motif (ITIM) and Immunoglobulin tail tyrosine (ITT)-like motif, which are responsible to transmit negative signal when binding with its two ligands CD155 and CD112 in a cell-intrinsic way (18). Previous studies have shown that TIGIT expressed on effector T cells as negative feedback to maintain T cell self-tolerance, while on Treg as an inherent suppressive regulation for effector Th1 and Th17 (15, 19). Accumulating evidence have proved that TIGIT are involved in the development of autoimmunity, cancer and chronic infection (15). In sepsis, the expansion of TIGIT^+^ Treg are related to post sepsis immunosuppression (13, 20), while anti-TIGIT antibody treatment improved survival in cancer mice with sepsis (21). However, since hyperinflammation and immunosuppression occur sequentially or concurrently or take turns during the development of sepsis (6), the role of TIGIT on the acute stage of sepsis remains enigmatic.

In this study, TIGIT knockout mice (TIGIT^-/-^) and anti-TIGIT antibody were employed to interrogate whether TIGIT is involved in bacteria clearance at the acute phase of experimental sepsis. Our results showed that the expression of TIGIT and its ligand CD155 were upregulated in CLP-induced septic mice. CD4^+^ T cell immunity against polymicrobial infection during CLP-induced sepsis was enhanced by genetic ablation or antibody blockade of TIGIT. Bacterial load was detected lower in the peritoneal lavage fluid (PF) or liver. As a result, TIGIT deficiency or blockade protected mice from sepsis. These results suggest that TIGIT could be an important target for sepsis immunotherapy at the acute stage of sepsis.

## Results

### Expression patterns of TIGIT and CD155 in splenocytes during acute sepsis

**Figure 1:**
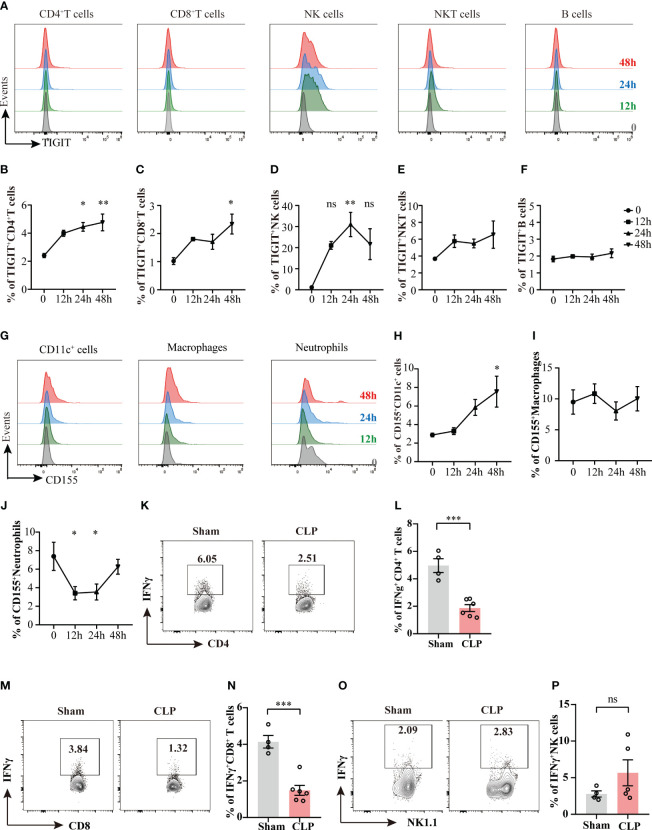
The kinetics of TIGIT and CD155 expression in splenocytes during acute phase of sepsis. Splenocytes were harvested on 12h, 24h, and 48h after CLP surgery and TIGIT and CD155 expression on splenic immune cells was measured. **(A)** Representative flow cytometry histograms showing TIGIT expression on different lymphocyte populations at 12h (green, n=5), 24h (blue, n=5) and 48h (red, n=5) in comparison to sham at 0h (grey, n=3). Summarized frequencies of splenic TIGIT^+^CD4^+^ T cells **(B)**, TIGIT^+^CD8^+^ T cells **(C)**, NK cells **(D)**, NKT cells **(E)** and B cells **(F)** at various time points during acute sepsis were presented. **(G)** Representative flow cytometry histograms showing CD155 expression on different myeloid cell populations. Summarized frequencies of splenic CD155^+^CD11c^+^ cells **(H)**, CD155^+^macrophages **(I)** and CD155^+^neutrophils **(J)** at various time points. Representative flow cytometry counter plots **(K)** and statistical graphs **(L)** of IFN-γ expression in CD4+ T cells from sham (n=4) or CLP (n=6) mice 24h post CLP. Representative flow cytometry counter plots **(M)** and statistical graphs **(N)** of IFN-γ expression in CD8+ T cells from sham or CLP mice 24h post CLP. Representative flow cytometry counter plots **(O)** and statistical graphs **(P)** of IFN-γ expression in NK cells from sham or CLP mice 24h post CLP. Data are means ± SEM. Comparison between groups were analyzed using one-way ANOVA and Dunnett’s multiple comparisons test and Student’s *t*-test. *p < 0.05, **p < 0.01, ***p < 0.001; *ns*, not significant.

Previous studies have found that co-inhibitory molecules, such as PD-1, CTLA-4, LAG-3, VISTA, are upregulated during sepsis (22, 23), we then queried the expression of TIGIT and its ligand CD155 upon sepsis insult. To address this issue, the expression levels of TIGIT on different lymphocytes were measured at different time points during the acute phase of sepsis (**Figure 1A**). TIGIT was upregulated as early as 24h after CLP induction in CD4^+^ T cells and NK cells compared with sham group harvested at 0 hour (**Figures 1B, D**). TIGIT expression in CD8^+^ T cells was elevated at 48h post CLP (**Figure 1C** **).** No significant increases were observed in NKT cells and B cells across these detected time points (**Figures 1E, F**). In addition, the dynamic change of CD155 expression on myeloid cells was also measured by flow cytometry (**Figure 1G**). CD155 expression on CD11c^+^ dendritic cells were induced significantly at 48h post CLP in comparison to sham controls (**Figure 1H**). CD155 on macrophages was not different between CLP and sham by 48h (**Figure 1I**). However, CD155 expression on neutrophils were dramatically reduced at both 12h and 24h post CLP (**Figure 1J**), suggesting that the expression pattern of CD155 among myeloid cells was relatively more heterogeneous and flexible during sepsis onset. In addition, we further compared IFN-γ expression between sham mice and CLP mice 24h post CLP. There is a significant decrease of IFN-γ production in in both CD4^+^ and CD8^+^ T cells from CLP mice when compared sham mice (**Figures 1K–N**). However, we did not observe change of IFN-γ production by NK cells (**Figures 1O, P**). Therefore, it suggests that T cell effector function is impaired during sepsis. Taken together, these data uncovered that TIGIT was predominantly upregulated on CD4^+^ T cells, CD8^+^ T cells and NK cells and increased TIGIT suppressed T cell function at the initiated stage of sepsis.

### TIGIT and CD155 expression are upregulated in experimental septic mice

**Figure 2:**
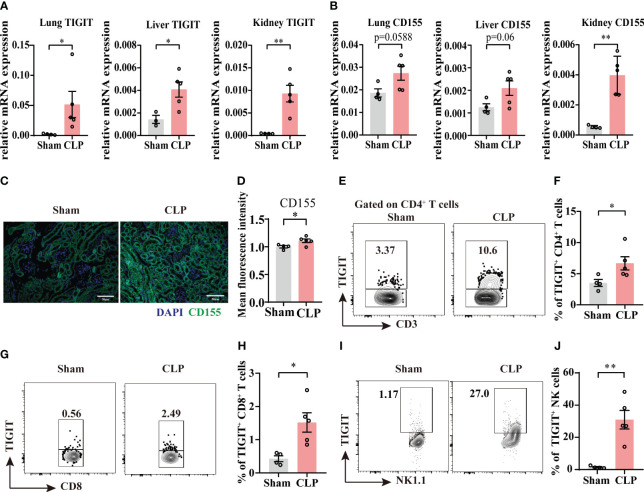
The expression of TIGIT and CD155 are upregulated in sepsis mice. Relative mRNA expression level of TIGIT **(A)** and CD155 **(B)** in the lung, liver and kidney of sham and experimental sepsis mice. **(C)** Representative immunofluorescence images co-staining of DAPI (blue), CD155 (green) and merged images on kidney section from sham and CLP mice. Magnification: 400 ×; scale bar, 50 μm. **(D)** Statistical graph showing the mean fluorescence intensity of CD155 in kidney section from sham and CLP mice. **(E-J)** TIGIT expression on spleen CD4^+^, CD8^+^ T cells and NK cells was measured by flow cytometry. **(E)** Representative flow cytometry counter plots showing TIGIT expression on CD4^+^ T cells from spleen of sham and CLP mice. **(F)** Summarized frequencies of TIGIT^+^CD4^+^ T cells from spleen were presented. **(G)** Representative flow cytometry counter plots showing TIGIT expression on CD8^+^ T cells from spleen of sham and CLP mice. **(H)** Summarized frequencies of TIGIT^+^CD8^+^T cells from spleen were presented. **(I)** Representative flow cytometry counter plots showing TIGIT expression on NK cells from spleen of sham and CLP mice. **(J)** Summarized frequencies of TIGIT^+^ NK cells from spleen were presented. Data are means ± SEM. Comparison between sham (n=4) and CLP (n=5) were analyzed using Student’s *t*-test. *p < 0.05, **p < 0.01; *ns*, not significant.

According to our observations that TIGIT was upregulated 24h after sepsis insult in **Figure 1**, a 24h cecal ligated and punctured mouse model was used in this study. In CLP group, the relative mRNA expression of TIGIT in lung, liver and kidney were higher than that of Sham group (**Figure 2A**). Moreover, CD155 expression is significantly increased in the kidney from sepsis mice relative to Sham mice, while in lung and liver, an increased trend was shown (**Figure 2B**). Indeed, previous study has proved that CD155 is constitutively expressed on renal tubular cells but not glomerular cells (24) Here, immunofluorescence staining showed the similar location of CD155 in kidney (**Figure 2C**) and CD155 expression level was increased after CLP insult as indicated by mean fluorescence intensity (MFI) (**Figure 2D**).

To further elucidate TIGIT expression on T cells and NK cells during acute sepsis, splenocytes from septic and sham mice were evaluated by flow cytometry. Our data showed that TIGIT expression was markedly increased on both total CD4^+^ T cells and CD8^+^ T cells during sepsis (**Figures 2E–H** **).** Moreover, a significant increase of TIGIT in NK cells was also observed as expected (**Figures 2I, J**). These observations were in accordance with previous study that TIGIT expression is augmented at the initiation of T cell activation under inflammatory conditions (25, 26). According to the above results, we hypothesized that TIGIT may regulate T cells responses during acute sepsis.

### TIGIT deficiency protects mice from organ injury

**Figure 3:**
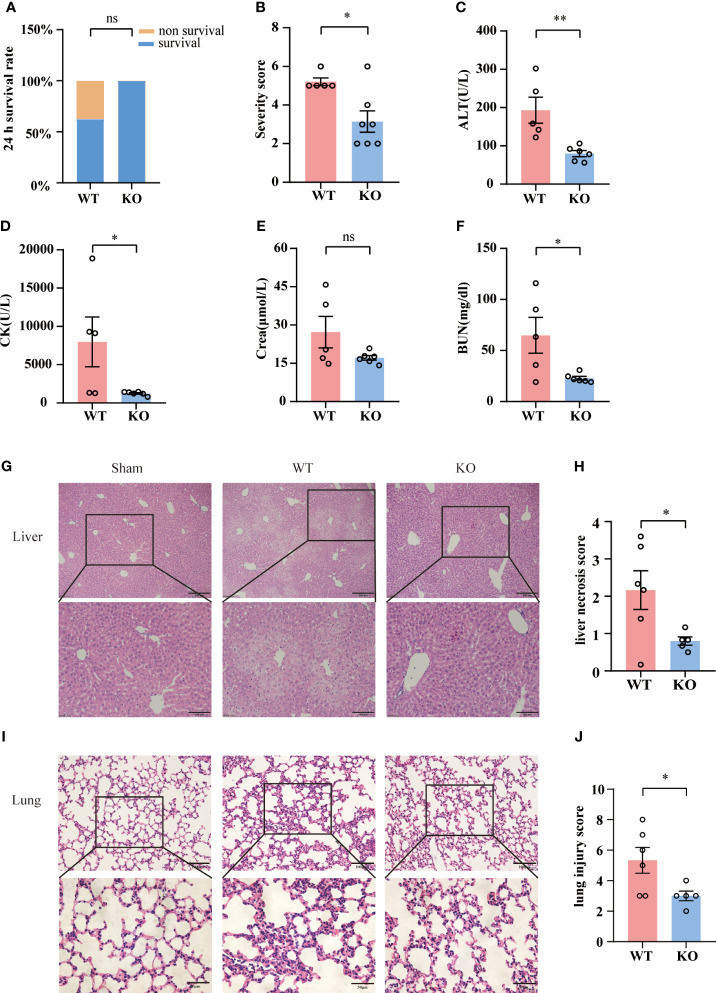
TIGIT deficiency confers protection from CLP-inducing sepsis. **(A)** Survival rate was recorded 24 hours post CLP. After CLP surgery, TIGIT^-/-^ mice (n=7) demonstrated an elevated trend of 24-hour survival rate relative to WT (n=8) mice. **(B)** At the time of 24-hour, severity score was evaluated to assess clinical symptoms blindly. **(C–F)** Serum concentrations of ALT, CK, creatinine (Cr) and BUN were measured in WT and TIGIT^-/-^ mice. **(G)** Representative H&E staining images of liver from WT and TIGIT^-/-^ mice, depicting more severe necrosis areas and **(H)** liver necrosis score were shown. Magnification: 100 ×; scale bar: 200 μm (top). Magnification: 200 ×; scale bar, 100 μm (bottom). **(I)** Representative H&E staining images of lung from WT and TIGIT^-/-^mice and **(J)** lung injury score. Magnification: 200 ×; scale bar: 100 μm (top). Magnification: 400 ×; scale bar, 50 μm (bottom). Comparison are means ± SEM. Data between WT (n=5) and TIGIT^-/-^ (n=6) were analyzed using Student’s *t-*test. *p < 0.05, **p < 0.01; *ns*, not significant.

To access the impact of TIGIT deficiency on acute sepsis, we employed wild-type (WT) or TIGIT^-/-^ mice for CLP. Although 24-hour survival rate revealed no significant differences between WT and TIGIT^-/-^ mice, there was no death in TIGIT^-/-^ mice while 37.5% of WT mice died 24 hours after CLP operation (**Figure 3A**). In line with mortality, TIGIT^-/-^ mice displayed significantly lower disease severity score than WT mice (**Figure 3B**). We further evaluated organ injury through detecting the levels of alanine transaminase (ALT), creatine kinase (CK), creatinine (Cr) and blood urea nitrogen (BUN) in serum harvested from mice 24 hours after CLP. TIGIT^-/-^ mice have significantly reduced serum level of ALT, CK and BUN compared with WT mice, while serum level Cr was comparable (**Figures 3C–F**). Histological analysis by H&E staining showed that TIGIT deficiency attenuated necrosis areas of liver tissue (**Figures 3G, H**) and reduced lung damage (**Figures 3I, J**), as indicated by less infiltration of inflammatory cells in alveolar wall or airspace. These results confirmed that TIGIT deficiency conferred protective effect in mice under CLP induced sepsis.

### TIGIT deficient promotes bacterial clearance in sepsis

**Figure 4:**
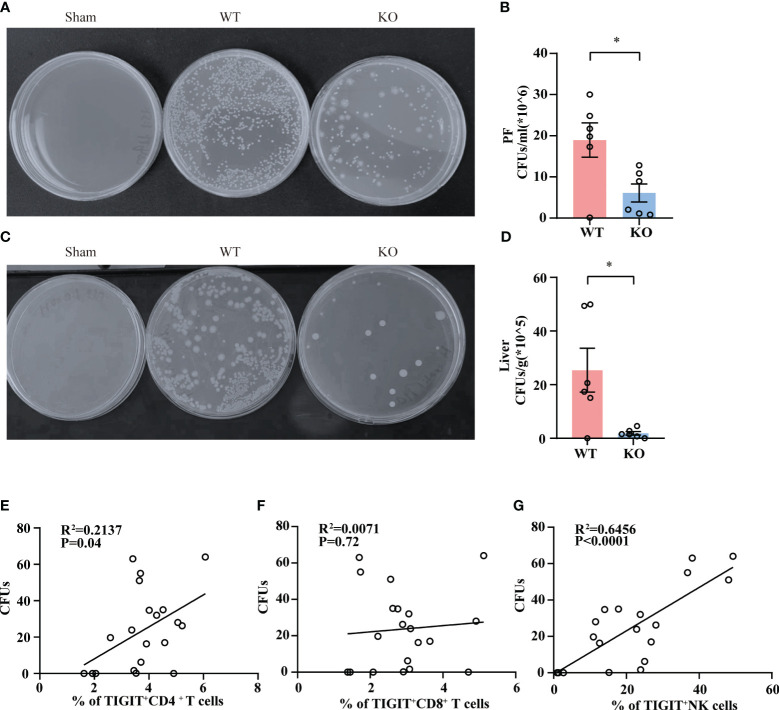
TIGIT deficiency promotes bacterial clearance. Twenty-four hours post CLP, WT and TIGIT^-/-^ mice were sacrificed and PF and liver tissues were obtained for bacterial culture. CFUs were counted after 24 hours and data were presented as the number of CFUs per ml for PF **(A, B)** and CFUs per gram for liver **(C, D)**. **(E)** Correlations between the percentage of TIGIT^+^CD4^+^ T cells and PF CFUs. **(F)** Correlations between the percentage of TIGIT^+^CD8^+^ T cells and PF CFUs. **(G)** Correlations between the percentage of TIGIT^+^NK cells and PF CFUs. Spearman’s R square and a regression line are indicated. (n=20) Data are means ± SEM. Comparison between WT (n=6) and TIGIT^-/-^(n=6) were analyzed using Student’s *t*-test. *p < 0.05; *ns*, not significant.

To clarify whether TIGIT^-/-^ mice experienced a milder injury as a result of improved bacterial clearance efficiency, we detected the bacterial load in abdominal cavity and liver tissue in WT and TIGIT^-/-^ mice. As shown in (**Figures 4A, B** **),** TIGIT^-/-^ mice exhibited a significantly reduced bacterial load in PF during sepsis. In addition, markedly less bacterial load in liver tissue was also observed in TIGIT^-/-^ mice relative to WT mice (**Figures 4C, D** **).** In addition, we observed a positive correlation between the percentage of TIGIT^+^CD4^+^ T cells and bacterial load in PF (**Figure 4E**). There was also a correlation between the percentage of TIGIT^+^ NK cells and bacterial load in PF (**Figure 4F**). However, the percentage of TIGIT^+^ CD8^+^ T cells showed no correlation with bacterial load in PF (**Figure 4G**). These data indicated that TIGIT deficiency improved bacterial clearance capability in septic mice, leading to relieved injury during sepsis.

### TIGIT deficiency promotes CD4^+^ T cell effector function and innate immunity activation in sepsis

Since sepsis mice exhibited elevated level of TIGIT in T cells, we tried to figure out the differences in effector cytokine level between TIGIT^+^ and TIGIT^-^ T cells. Under Sham condition, both TIGIT^+^ CD4^+^ T cell and CD8^+^ T cells exhibited significantly higher expression of IFN-γ compared to TIGIT^-^ CD4^+^ T cells and CD8^+^ T cells (**Supplementary Figures 1A–D**). In addition, under CLP condition, TIGIT^+^ CD4^+^ T cells have significantly higher expression level of IFN-γ than their TIGIT^-^ counterparts, while TIGIT^+^ CD8^+^ T cells only show an increasing trend for IFN-γ expression (**Supplementary Figures 1A–D**). Next, we utilized public RNA-seq dataset (GSE216902) to find out the relationship between TIGIT mRNA expression and cytokine level in sepsis patients of acute phase. When 37 acute sepsis patients on day 1 were grouped into TIGIT^hi^ and TIGIT^lo^ according to the transcriptional level of TIGIT, we found that patients with high expression of TIGIT had a markedly increased level of IFN-γ compared to those with low TIGIT expression (**Supplementary Figure 1E**). Kyoto Encyclopedia of Genes and Genomes (KEGG) enrichment also showed that differentially expressed genes (DEGs) of TIGIT^hi^ compartments were enriched in Th17 cell differentiation and Th1 and Th2 cell differentiation (**Supplementary Figure 1F**). These results confirmed that TIGIT expression was relevant to more functionally T cell activation under both steady condition and inflammatory condition.

**Figure 5:**
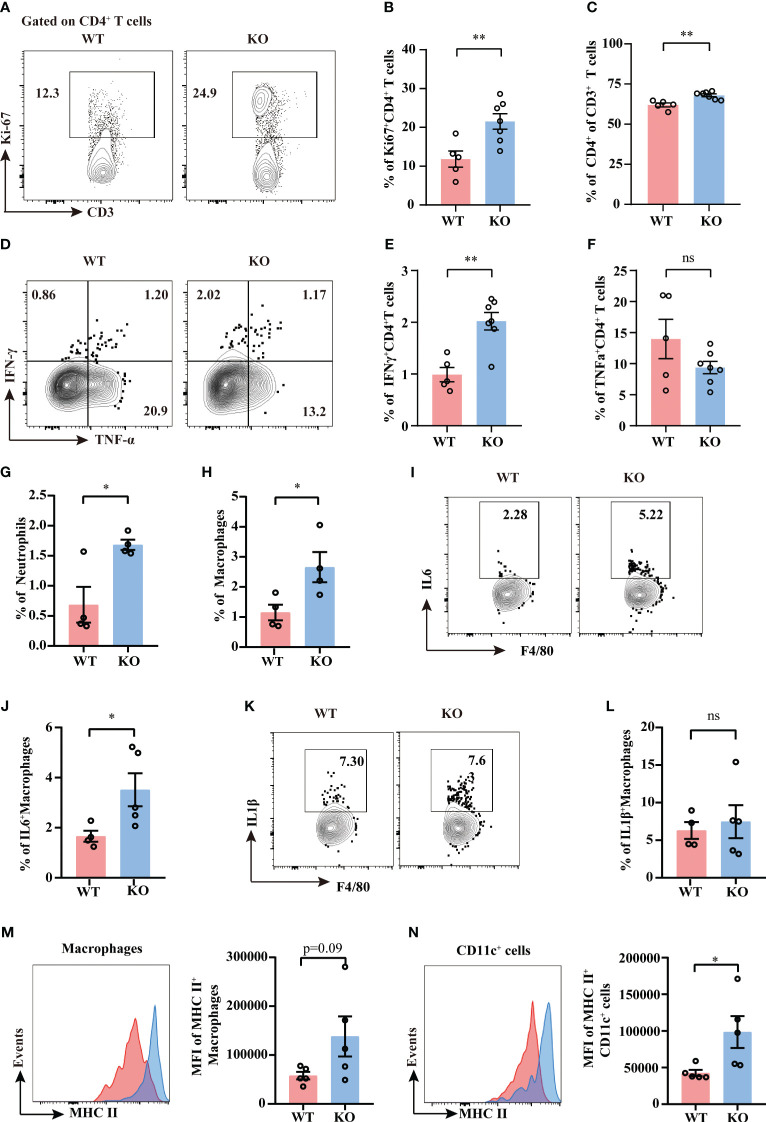
TIGIT deficiency promotes CD4^+^ T cells proliferation and activation. Splenocytes from septic mice were isolated and stimulated with PMA, ionomycin and BFA for 4.5 hours to assess T cell cytokine production while stimulated with LPS and BFA for 5 hours to access macrophage production, and then analyzed by flow cytometry. **(A)** Representative flow cytometry counter plots of Ki67 expression in CD4^+^ T cells from spleen of TIGIT WT and TIGIT^-/-^ mice underwent CLP and **(B)** analysis of the percentage were shown. **(C)** Statistical graphs showing the percentage of CD4^+^ T cells among CD3^+^ T cells. Representative flow cytometry counter plots **(D)** and statistical graphs of IFN-γ **(E)** and TNF-α **(F)** expression in CD4^+^ T cells from WT or KO septic mice. Data are means ± SEM. Comparison between WT (n=5) and TIGIT^-/-^ (n=7) were analyzed using Student’s t-test. **p < 0.01; ns, not significant. Statistical graphs showing the percentage of neutrophils **(G)** and macrophages **(H)** from spleen of WT and TIGIT^-/-^ mice underwent CLP. **(I)** Representative flow cytometry counter plots of IL-6 expression in macrophages from spleen of WT or TIGIT^-/-^ septic mice. (J) Statistical graphs of IL-6 expression in macrophages from spleen of WT or TIGIT^-/-^ septic mice. (K) Representative flow cytometry counter plots of IL-1β expression in macrophages from spleen of WT or TIGIT^-/-^ septic mice. (L)Statistical graphs of IL1β expression in macrophages from spleen of WT or TIGIT^-/-^ septic mice. (M) Representative flow cytometry histogram and statistical graph showing the mean fluorescence intensity (MFI) of MHC II+ macrophages from spleen of WT and TIGIT^-/-^ mice underwent CLP. **(N)** Representative flow cytometry histogram and statistical graph showing the MFI of MHC II^+^CD11c^+^ cells from spleen of WT and TIGIT^-/-^ mice underwent CLP. Data are means ± SEM. Comparison between WT (n=4/5) and TIGIT^-/-^ (n=5) were analyzed using Student’s *t*-test. *p < 0.05; *ns*, not significant.

It is well demonstrated that TIGIT conducts inhibitory regulation upon activation. To further elucidate how TIGIT deficiency reduced organ damage and bacterial load in CLP-induced sepsis, we assessed the proliferation and effector function of splenic T cells and NK cells from septic mice. Albeit TIGIT deficiency showed no influence on the percentages of total CD4^+^ and CD8^+^T cells (**Supplementary Figures 2A, B**), our data showed that the percentage of Ki-67^+^ CD4^+^ T cells was significantly higher in TIGIT^-/-^ group than WT group, which coincided with increased proportion of CD4^+^ in CD3^+^ T cells in TIGIT^-/-^ mice compared to WT mice (**Figures 5A–C**). In terms of proinflammatory cytokines production, the expression of IFN-γ in CD4^+^ T cells was upregulated in TIGIT^-/-^ mice compared to WT mice (**Figures 5D, E**), while there was no significant difference concerning the percentages of TNFα^+^ T cells (**Figure 5F**). However, our results revealed no difference in Ki-67 and IFN-γ expression in CD8^+^ T cells between WT and TIGIT^-/-^ mice (**Supplementary Figures 2C–F** **).** Unexpectedly, Ki-67 and IFN-γ levels were not different in NK cells (**Supplementary Figures 2H–K**). In addition, the proportion of Foxp3^+^ CD4^+^ cells was comparable between the two groups (**Supplementary Figures 2L, M**). The above results suggest that TIGIT deficiency promotes CD4^+^ T cells expansion and effector function during sepsis.

Given that CD4^+^ T cell-derived IFN-γ has long been identified as an important player in providing activating signals to innate immune system (8), we next investigate the innate immune profiles induced by TIGIT deficiency. TIGIT^-/-^ mice showed increased percentages of splenic neutrophils and macrophages as compared to WT mice (**Figures 5G, H**). To further study the function of macrophages and dendritic cells, intracellular cytokine production and MHC class II molecule expression were measured by flow cytometry. IL-6 expression was upregulated in macrophages from TIGIT^-/-^ mice when compared to that from WT mice (**Figures 5I, J**), whereas IL-1β expression was similar between the two groups (**Figures 5K, L**). There was an increasing trend for the MFI of MHC II in macrophages (**Figure 5M**). MHC II expression in TIGIT^-/-^ dendritic cells was notably increased compared to WT cells (**Figure 5N**). These phenotypes of activated innate immunity may contribute to bacterial clearance. In summary, IFN-γ-producing Th1 cells with elevated proliferation and effector function exert crucial role in robust innate immune responses, which may account for efficient antimicrobial ability in TIGIT deficient mice.

### TIGIT blockade limits the severity of organ damage and bacterial infection

**Figure 6:**
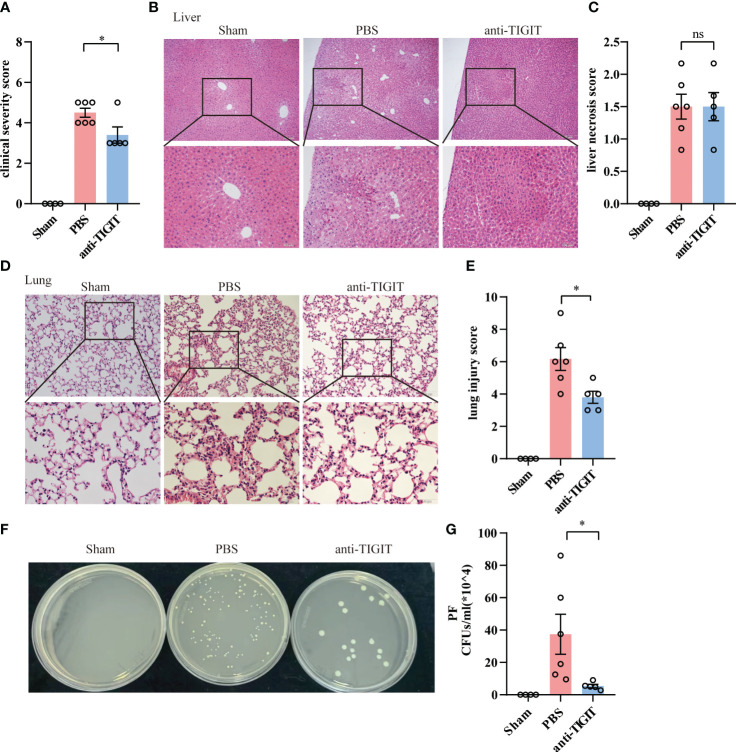
TIGIT blockade protects mice from CLP-induced sepsis. Mice treated with anti-TIGIT Ab or PBS 24 hours before CLP induction were sacrificed 24 hours after the surgery. **(A)** At the time of 24-hour, severity score was evaluated to assess clinical symptoms blindly. **(B)** Representative H&E staining images of liver from sham and septic mice treated with PBS or anti-TIGIT Ab and **(C)** liver necrosis score were shown. Magnification: 100 ×; scale bar: 200 μm (top). Magnification: 200 ×; scale bar, 100 μm (bottom). **(D)** Representative H&E staining images of lung and **(E)** lung injury score were shown. Magnification: 200 ×; scale bar: 100 μm (top). Magnification: 400 ×; scale bar, 50 μm (bottom). **(F, G)** CFUs in PF from sham and sepsis mice treated with PBS or anti-TIGIT Ab were counted and data were presented as the number of CFUs per ml. Data are means ± SEM. Comparison between PBS (n=6) and anti-TIGIT (n=5) were analyzed using Student’s *t*-test. *p < 0.05; *ns*, not significant.

Given the role of TIGIT in negative regulation of CD4^+^ T cell function in the context of acute sepsis, we determined whether TIGIT blockade could lead to similarly relieved prognosis shown in TIGIT deficient mice. Since previous studies has proved that TIGIT blocking treatment after CLP failed to improve survival and organ damage in septic mice with naïve background (21, 27), we next wanted to explore whether TIGIT blockade prior to CLP insult has effect on sepsis. WT mice were randomized to receive either anti-TIGIT antibody or PBS as control, followed by CLP induction after 24h. The pretreatment with anti-TIGIT Ab prevented CLP-induced sepsis based on a significant decrease in clinical severity score (**Figure 6A**). Although histological images showed the degree of liver injury was comparable between anti-TIGIT group and control (**Figures 6B, C**), lung injury was partially restored by administration of anti-TIGIT Ab (**Figures 6D, E**). In addition, colony-forming units (CFUs) in PF was counted to further quantify the effects of TIGIT blockade on the bacterial clearance capacity. Our results revealed a significant reduction in bacterial load in mice received TIGIT blocking treatment when compared to control mice (**Figures 6F, G**). Collectively, our data suggest that TIGIT blockade attenuates organ damage and infection in sepsis.

### TIGIT blockade facilitates T cell function and innate responses

**Figure 7:**
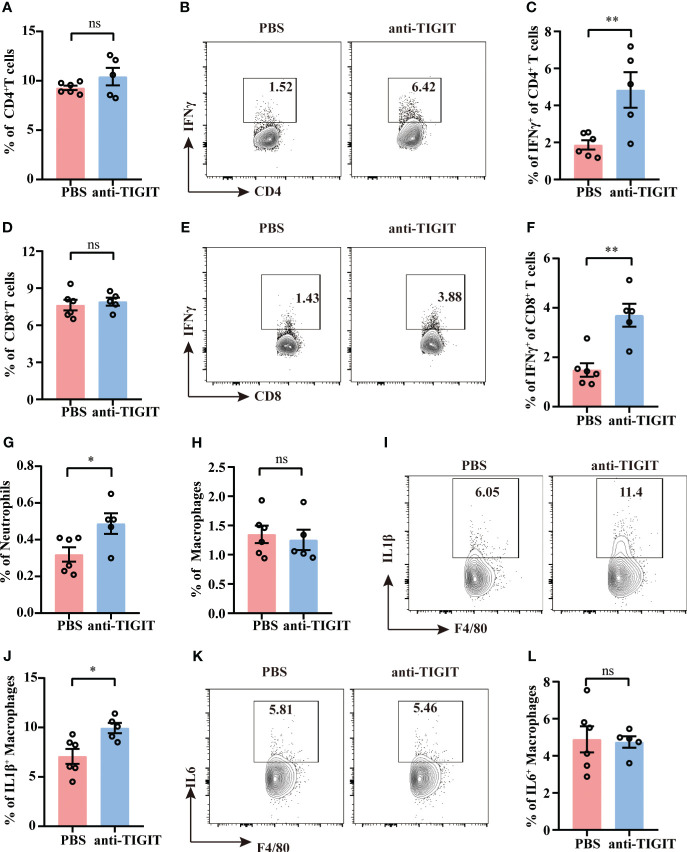
TIGIT blockade elevates IFN-γ production by T cells. Sepsis model induction and treatment as well as splenocyte isolation and stimulation were performed as described in **Figures 5**, **6**. **(A)** Statistical graphs showing the percentage of CD4^+^T cells of spleen from septic mice received PBS or anti-TIGIT Ab injection in advance. Representative flow cytometry counter plots **(B)** and statistical graphs **(C)** showing the percentage of IFN-γ expression in CD4^+^ T cells. **(D)** Statistical graphs showing the percentage of CD8^+^T cells of spleen from septic mice received PBS or anti-TIGIT Ab injection in advance. Representative flow cytometry counter plots **(E)** and statistical graphs **(F)** of IFN-γ expression in CD8^+^ T cells. Statistical graphs showing the percentage of macrophages **(G)** and neutrophils **(H)** of spleen from septic mice received PBS or anti-TIGIT Ab injection in advance. **(I)** Representative flow cytometry counter plots of IL-1β expression in macrophages of spleen from septic mice received PBS or anti-TIGIT Ab injection. **(J)** Statistical graphs of IL-1β expression in macrophages of spleen from septic mice received PBS or anti-TIGIT Ab injection. **(K)** Representative flow cytometry counter plots of IL6 expression in macrophage of spleen from septic mice received PBS or anti-TIGIT Ab injection. **(L)** Statistical graphs of IL-6 expression in macrophages of spleen from septic mice received PBS or anti-TIGIT Ab injection. Data are means ± SEM. Comparison between PBS (n=6) and anti-TIGIT (n=5) were analyzed using Student’s *t*-test. *p < 0.05, **p < 0.01; *ns*, not significant.

Despite the percentages of total CD4^+^ T cells and CD8^+^ T cells were not changed between mice injected with anti-TIGIT Ab and mice with PBS (**Figures 7A, D**), TIGIT blockade showed a marked upregulation of IFN-γ both in CD4^+^ T cells and CD8^+^T cells (**Figures 7B, C, E, F**), which is consistent with improvement of CD4^+^ T cell effector function in TIGIT deficiency. To further investigate the effects of blocking TIGIT on anti-infectious immunity, innate immune profiles were also depicted. According to our data, mice received anti-TIGIT Ab exhibited increased percentage of splenic neutrophils as compared to PBS control mice (**Figure 7G**), but the percentage of macrophages was similar in both groups (**Figure 7H**). In terms of innate immune cytokines produced by macrophages, we observed an elevated expression of IL-1β in mice received anti-TIGIT Ab (**Figures 7I, J**), whereas the expression of IL-6 was comparable (**Figures 7K, L**). Taken together, these findings proved that TIGIT blockade promoted T cell cytokine production and mobilized innate immunity during sepsis.

## Discussion

Emerging studies have suggested the engagement of coinhibitory receptors in dysfunctional T cell responses during sepsis (28, 29). Therapeutic methods targeting these coinhibitory receptors improve host resistance to infection and ameliorate sepsis morbidity and mortality (28, 30, 31). In the present study, we found that TIGIT is significantly upregulated in CD4^+^ and CD8^+^ T cells as well as NK cells in CLP challenged mice. TIGIT deficiency enhanced CD4^+^ T cell effector function and facilitated bacterial clearance during CLP induced-sepsis, resulting in decreased organ injury. These findings suggest that TIGIT may be a promising target for the treatment of sepsis.

It is now recognized that immunosuppression existing in sepsis is a predisposing factor in the paralyzed ability to remove pathogens and increased susceptibility of patients to secondary infections and mortality (6). When the initial inflammatory reactions triggered by infection are excessive in patients with sepsis and result in tissue damage and organ failure, anti-inflammatory responses are induced concurrently, and the persistence of the anti-inflammatory milieu promotes sustained immunosuppression (13). Sepsis-induced immunosuppression is related to the release of anti-inflammatory cytokines, expansion of immune suppressor cells, effector immune cell death, and co-inhibitory molecular expression (6). PD-1/PD-L1 pathway, for example, play an essential role in sepsis-induced immunosuppression through promoting T cells death and exhaustion, impairing its proliferation ability and inhibiting its proinflammation cytokine production (32, 33). In this study, we found that T cell function was impaired in acute sepsis as indicated by a significant decrease of IFN-γ production. Following CLP, the expression of TIGIT and CD155 in liver, lung and kidney tissues were increased. Further analysis showed that there was a significantly increased TIGIT expression on both T cells and NK cells 24 hours after CLP challenge, in accordance with published study (34) that TIGIT expression on T cells was upregulated in septic patients. CD155 is expressed on monocytes, dendritic cells and a variety of nonhematopoietic cell types (15). Previous study proved that TIGIT transduced suppressive signal to prevent dendritic cell maturation through combination with its ligand CD155 (17). Here, we found that CD155 expression on CD11c^+^ cells were also induced, indicating a potential role of TIGIT signaling pathway on the development of sepsis immunosuppression regulated by T cells.

Served as negative feedback on T cell, TIGIT conducts inhibitory signal through its intrinsic function or binding to its ligand CD155 (25, 35). The inhibitory signaling by phosphorylation of the tyrosine residue in the ITIM motif or the ITT-like motif, could result in the binding of cytosolic adaptor growth factor receptor-bound protein 2 (Grb2) and following recruitment of SH2 domain-containing inositol-5-phosphatase 1 (SHIP1), which mediates the inhibition of PI3K/AKT and MAPK/ERK pathway (15, 35, 36). In addition, phosphorylated ITT-like motif binds β-arrestin2 to TIGIT and recruits SHIP1 to limit TRAF6/NF-kB signaling (37). The abovementioned suppression of signaling pathways play crucial role in decreased Th1 and Th17 cytokine production (15). In addition, previous studies have shown that TIGIT expression on Tregs was associated with superior suppressive ability and lineage stability (38) and had been identified to selectively suppress Th1 and Th 17 responses and favored Th2 immunity and IL10 production (15, 19). In essence, TIGIT expression was induced upon stimulated with anti-CD3 and anti-CD28 *in vitro* and reached a peak at 24 hour and then decreased over time (18). Previous studies also showed that TIGIT-expressing T cells possessed a functionally proinflammatory profile and memory phenotype in context of experimental acute kidney injury, donor-specific hyperresponsiveness (DSH) and systemic lupus erythematosus (SLE) (39–41). TIGIT^+^ CD8^+^ T cells were suggested to maintain an intrinsic capacity to kill targets in HIV-infected individuals instead of exhibiting an exhausted function (42). Our data showed that TIGIT^+^ CD4^+^ or TIGIT^+^ CD8^+^ T cells possessed more productive cytokine-secreted capacity, conforming the activated and proinflammatory phenotypic feature of TIGIT^+^ T cell compartments. Transcriptomic analysis also revealed that the enriched DEGs of TIGIT^hi^ PBMC samples were involved in Th17, Th1 and Th2 cell differentiation, suggesting that TIGIT expression is related to T cell differentiation upon activation. These observation supports the above standpoint of functionally activated status of TIGIT-expressing T cells. Therefore, we identified TIGIT as a marker of early T cell activation and TIGIT^+^ T cells possessed a proinflammatory trait at the initiated stage of sepsis.

Although TIGIT deficiency or TIGIT blockade led to increased T cell proliferation, robust cytokine production and degranulation (39, 43, 44), murine models of different disease backgrounds showed various responses to the loss of TIGIT signaling pathway. Blocking TIGIT by antibody or genetic knockout enhanced NK cell activation and aggravated liver injury in a poly I:C/D-GalN-induced model of acute hepatitis (26). However, the absence of TIGIT protected mouse from Dextran Sodium Sulfate (DSS)-induced colitis through the regulating IL17A-producing tissue-resident memory T cells (45). In ischemia reperfusion (IR) and cisplatin AKI models, TIGIT regulated T cell responses and contributed to kidney pathological injury (39). In this research, TIGIT genetic deficient mice were employed for the first time in CLP-induced sepsis model. Our data uncovered that both of TIGIT^-/-^ mice and anti-TIGIT-treated mice were protected from CLP-induced sepsis as evidenced by lower bacterial load in PF and milder organ damage. In addition, we observed an advancement in proliferation and IFN-γ production in CD4^+^T cells when loss of TIGIT by genetic knockout or antibody treatment. However, the similar results were not observed in CD8^+^ T cells and NK cells. A positive correlation between TIGIT expression on CD4^+^T cells and bacterial burden in PF was also obtained. Therefore, we conformed that CD4^+^T cells exerted predominant influence on anti-infection responses when loss of TIGIT.

It is reported that CD4^+^ T cells are central for activating proinflammatory macrophages through releasing cytokines, such as IFN-γ (8). Activated macrophages augment pro-inflammatory cytokine release and class II MHC expression in addition to phagocytosis to fight against infection (46). Further, it has been proven that CD4^+^T is involved in the activation of early-responding immune cells in sepsis (6). Therefore, we characterized the profiles of neutrophils, macrophages and dendritic cells in TIGIT^-/-^ mice under sepsis challenge. Our results revealed that innate immune responses were mobilized in terms of increased neutrophil and macrophage proportions when loss of TIGIT. Moreover, blocking TIGIT signaling resulted in elevated inflammatory cytokine production and antigen presentation capacity in macrophages and dendritic cells. In this study, TIGIT modulated CD4^+^ T cell immunity against bacterial infection during sepsis. TIGIT deficiency or blockade enhanced CD4^+^ T cell function and thus promoted bacterial clearance.

It has been shown that TIGIT contributes to the development of sepsis in the context of preexisting malignancy (21), and a similar survival benefit of TIGIT blockade was also seen as results of lymphopenia reversion. However, in sepsis with an immunologically experienced background, TIGIT blockade in septic mice that had received pathogen exposure showed a deteriorative 7-day survival because of apoptosis of memory T cells and decreased cytokine-producing T cells (27). For dynamic characterization of hyperactivated inflammation and immune paralysis, the role of TIGIT in sepsis could be varied for different contexts and stages of diseases. To this regard, further study is required to fully elucidate the underlying mechanism of TIGIT during sepsis. To conclude, we report that TIGIT and CD155 are upregulated in acute sepsis and the elevated expression of TIGIT is associated with T cell activated and proinflammatory profiles. Loss of TIGIT augments CD4^+^ T cell proliferative and effector competence in bacteria clearance and thus confers protection in sepsis. Together, this study highlights that immunomodulatory therapy targeting TIGIT signaling may have potential value at the acute stage of sepsis.

## Methods and materials

### Animals

TIGIT^-/-^ mice and age- and gender-matched WT littermates were bred and maintained in a specific pathogen-free condition with constant temperature and 12 hours of light at Experimental Animal Center of Sun Yat-Sen University. Animal experiments were approved by the Ethics Committee of the Laboratory Animal Center of Sun Yat-Sen University and were carried out in accordance with the National Institutes of Health Guide for Care and Use of Animals.

### CLP

In order to induce polymicrobial infection, cecal ligation and puncture was performed in 8 to 10 weeks old male WT and TIGIT^-/-^ mice as previously described (47). In brief, mice were anesthetized and fixed on the surgical table. A 1-cm longitudinal midline incision was made in the lower abdomen and cecum was dissociated and exposed. Then half of the cecum below the ileocecal valve was ligated with a 3-0 silk. The tied cecum was then punctured using a 22-gauge needle twice and gently squeezed the perforated cecum to push the bacteria in cecum to spread before returning it back into the peritoneal cavity. The incision was closed in layers and all the mice received fluid resuscitation through subcutaneous administration 1ml of warm sterile saline. The Sham group was operated in the same way as CLP group except for the ligation and puncture. Twenty-four hours post CLP, all the mice were sacrificed, before which clinical severity score was evaluated based on the following six criteria: lethargy, piloerection, tremors, periorbital exudates, respiratory distress and diarrhea (48).

### Anti-TIGIT antibody treatment

For the TIGIT blocking Ab experiment, C57BL6/J WT mice were administrated 400μg anti-TIGIT antibody (clone 1G9, BioXcell) (21, 27) via intraperitoneal injection 24 hours in advance, followed by CLP surgery to induce similar level of sepsis as WT mice received PBS control. Twenty-four hours post CLP, all the mice were sacrificed for further analysis.

### Histopathology

Lung and liver tissues were flushed in PBS and immersed in 4% neutral paraformaldehyde solution overnight for fixation, followed by paraffin embedding. After removing the wax by descending concentrations of alcohol series, 4μm-thick sections of lung and liver were stained with hematoxylin and eosin (H&E) and captured by microscopy (Olympus BX63F, Japan) under bright field for subsequent analysis.

Liver necrosis score was performed on a five-point scale of 0-4 as mentioned previously (49):0, no liver necrosis; 1, single cell necrosis; 2, up to 30% necrosis; 3, 31-60% necrosis; and 4, more than 60% necrosis. Lung injury score was evaluated as described previously (50), which contained 4 subscores:1) hyperemia; 2) hemorrhage; 3) infiltration of neutrophils in the airspace or vessel wall; and 4) thickness of the alveolar wall/hyaline membrane formation. For each criterion: a semiquantitative scale was performed: 0, little injury; 1+, mild injury (<25% lung field); 2+, moderate injury (25%-50% lung field); 3+, severe injury (50%-75% lung field); and 4+, maximal injury (>75% lung field). Both of the tissue images were scored by two pathologists blindly.

### Immunofluorescence staining

Kidney paraffin sections (4mm) were first dewaxed and rehydrated, and then subjected to 15 min microwave oven heating in a citrate buffer (10 mM, pH 6.0) for antigen-retrieval. For CD155 detection, sections were incubated with AF488 conjugated anti-CD155 antibodies (1:200, Bioss, China, catalog #bs-2525R-AF488) at 4°C overnight. Sections were washed with phosphate-buffered saline (PBS) three times and subsequently counterstained with DAPI in mounting medium. Images were captured with a fluorescence microscope (Olympus).

### Serum enzyme

Blood samples were collected from mice 24 hours after surgery and placed at room temperature for 2 h. Then blood samples were centrifuged at 3,000 rpm for 10 minutes and supernatant serum was collected and stored at -80°C for further measurement. Serum ALT, AST, CK, Creatine, BUN were detected by Automatic Biochemical Analyzer (Mindray BS-240VET, China).

### Bacteria culture

Peritoneal lavage fluid was harvested by washing peritoneal cavity with 3 mL sterile PBS 24-hour post CLP. For liver bacterial load detection, fresh liver tissues were weighed and homogenized in 1 mL PBS. One hundred μL of PF or liver homogenates were well mixed followed by serial 10-fold dilution in PBS. Then the samples were coated on Luria Bertani (LB) agar plates overnight at 37°C in 5% CO_2_ and colony forming units were counted.

### PCR

Total RNA was extracted using AG RNAex Pro Reagent (Accurate Biotechnology, China) according to the manufacturer’s instructions. Then mRNA was reversely transcribed to cDNA using Evo M-MLV RT Master Mix (Accurate Biotechnology, China). Real time quantitative PCR assay was performed using SYBR^®^ Green Premix Pro Taq HS qPCR Kit (Accurate Biotechnology, China) and cDNA synthesis process was in accordance with the manufacturer’s instructions on Applied Biosystems™QuantStudio (Thermo Fisher Scientific, USA). Relative expression levels of genes were calculated using 2-ΔΔCT formula, with GAPDH as the internal reference. The Primer sequences are provided in the **Supplementary Table 1**.

### Flow cytometry

Spleen were homogenized into single-cell suspensions through gently smashing with a syringe plunger in PBS containing 1% FBS and filtered through a 70-μm cell strainer. After hemolysis with Red Blood Cell Lysis Buffer (Sigma, USA), splenocytes were stained with fluorescence-labeled antibodies for flow cytometry analysis.

For cell surface staining, cells were stained with antibodies against CD3(Alexa Fluor 700, clone# 17A2), CD8a(Pacific Blue, clone#53-6.7), NK1.1(PE/Cy7, clone#S17016D), B220(FITC, clone# RA3-6B2), TIGIT(PE/Dazzle™ 594, clone#1G9), CD11b(FITC, clone#M1/70), CD11c(PerCP/Cy5.5, clone#N418), Ly6G(APC/Cy7, clone#1A8), F4/80(BV421, clone#BM8, eBioscience), I-A/I-E(PE/Dazzle 594, clone#M5/114.15.2) and CD155(PE/Cy7, clone#TX56) at 4°C for 30 min and then washed with PBS twice. For lymphocyte intracellular cytokines staining, cells were stimulated with phorbol 12-myristate 13-acetate (50 ng/ml), ionomycin (500 ng/ml), and brefeldin A (5 mg/ml) (all from Sigma, USA) for 4.5 h, followed by permeabilization with BD Cytofix/Cytoperm™ Fixation and Permeabilization Solution (BD biosciences, USA) according to the manufacturer’s instructions. Then splenocyte suspensions were stained with antibodies against IFN-γ (PE, clone#XMG1.2), TNF-α (FITC, clone#MP6-XT22) at 4°C for 30 min. For macrophage intracellular cytokines staining, cells were stimulated with LPS (100ng/ml) and brefeldin A (5 mg/ml) for 5 hours. Cells were permeabilized with the fixation/permeabilization solution, stained with antibodies against IL6(PE, clone#MP5-20F3) and IL1β (PE/Cy7, clone# NJTEN3, eBioscience) at 4°C for 30 min. For Foxp3 and Ki67 staining, cells were permeabilized with the fixation/permeabilization solution (eBioscience, USA) followed by incubation with antibodies against Foxp3 (Alexa Fluor^®^ 488, clone#150D) and Ki-67(APC, clone#16A8). All the antibodies were purchased from BioLegend (USA). Flow cytometry was performed on BD FACS celesta (BD, USA) and data was analyzed using Flowjo Software.

### Statistics

Statistical analysis was performed using GraphPad Prism 8.0. Data are presented as mean ± SEM. Comparisons between different groups were conducted under *t* test or one way ANOVA followed by adjusted multiple comparison as appropriate. *p < 0.05, **p < 0.01, ***p < 0.001; *ns*, not significant.

## Data availability statement

The original contributions presented in the study are included in the article/**Supplementary Material**. Further inquiries can be directed to the corresponding author.

## Ethics statement

The animal study was approved by Ethics Committee of the Laboratory Animal Center of Sun Yat-Sen University. The study was conducted in accordance with the local legislation and institutional requirements.

## Author contributions

XZ: Data curation, Investigation, Visualization, Writing – original draft. HX: Data curation, Investigation, Writing – review & editing. SW: Data curation, Investigation, Writing – review & editing. TR: Investigation, Writing – review & editing. JC: Investigation, Writing – review & editing. YH: Writing – review & editing. NY: Conceptualization, Data curation, Funding acquisition, Supervision, Writing – original draft, Writing – review & editing.
