# Supplementary material for: TIGIT regulates CD4+ T cell immunity against polymicrobial sepsis
Source: Front Immunol. 2024 Mar 13;15:1290564. doi: 10.3389/fimmu.2024.1290564 (PMC10965661; doi:10.3389/fimmu.2024.1290564)
Supplement: Supplementary file 1 [file DataSheet_1.docx]

Supplementary Material

TIGIT regulates CD4^+^ T cell immunity against polymicrobial sepsis

Xuexin Zhong^1+^, Haiping Xie^1+^, Shuang Wang^1^, Tingting Ren^1^, Junlin Chen^2^, Yuefang Huang^2^, Niansheng Yang^*1^

*** Correspondence:** Niansheng Yang, MD, PhD: yangnsh@mail.sysu.edu.cn

# Supplementary Figures and Tables

## Supplementary Figures

­


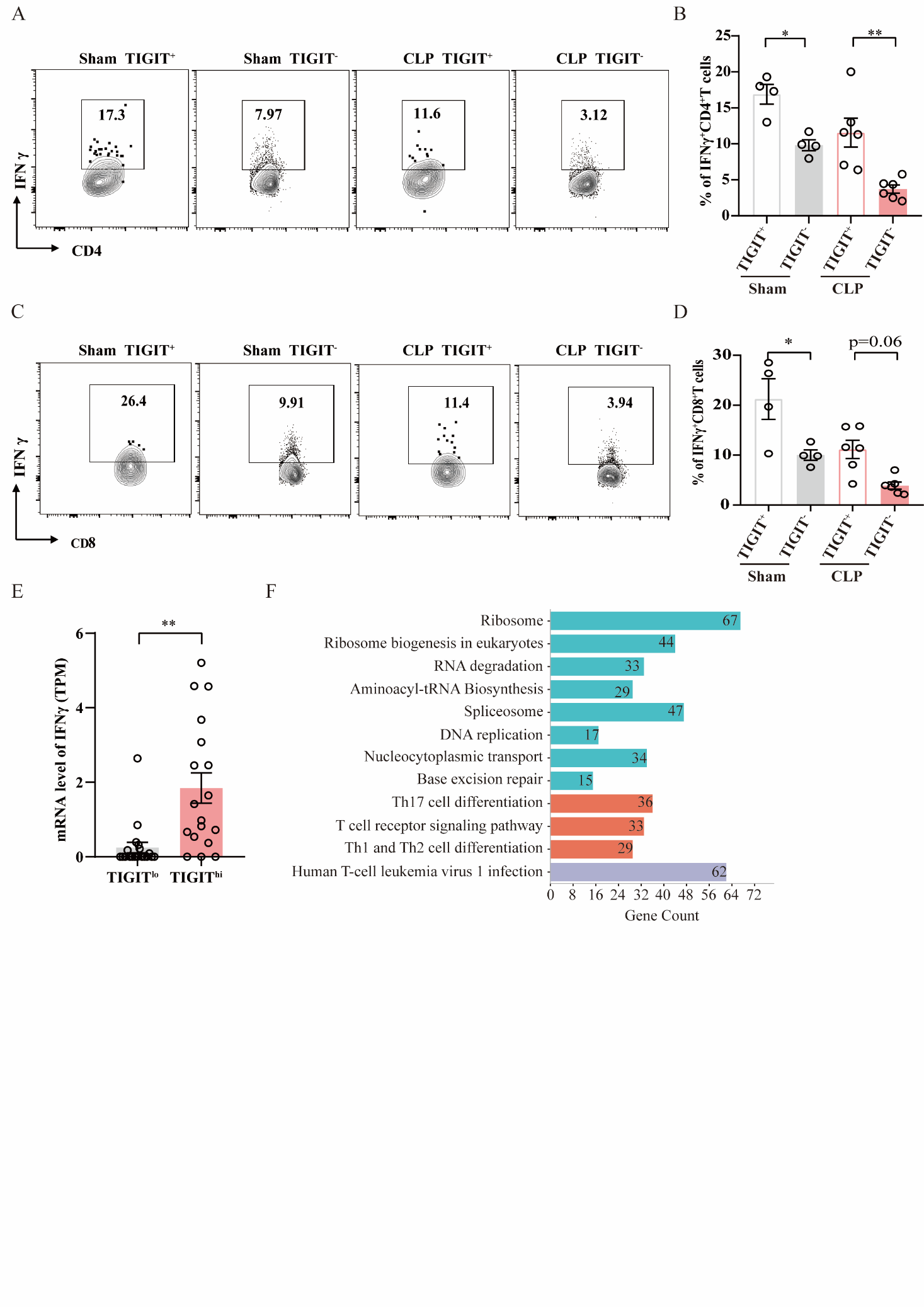


**Supplementary Figure 1. TIGIT^+^ T cells have enhanced cytokine production.** Splenocytes from sham and sepsis mice were isolated and stained with fluorescence-labeled antibodies and were analyzed by flow cytometry. **(A)** Representative flow cytometry counter plots of IFN-γ expression in TIGIT^+^ and TIGIT^-^ CD4^+^ T cells in CLP mice and control. **(B)** Statistical graphs showing the percentage of cellular cytokine IFN-γ of TIGIT^+^ and TIGIT^-^ CD4^+^ T cells in CLP mice and control. **(C)** Representative flow cytometry counter plots of IFN-γ expression on TIGIT^+^ and TIGIT^-^ CD8^+^ T cells in CLP mice and control. **(D)** Statistical graphs showing the percentage of cellular cytokine IFN-γ of TIGIT^+^ and TIGIT^-^ CD8^+^ T cells in CLP mice and control. Data are means ± SEM. Comparison between sham (n=4) and CLP (n=6) were analyzed using Student’s *t*-test and one-way ANOVA and Tukey's multiple comparisons test. **p* < 0.05, ***p* < 0.01; *ns*, not significant. High TIGIT expression is related to T cell differentiation based on PBMC transcriptomic data. RNA sequencing data was downloaded under accession number GSE216902 from the National Center for Biotechnology Information (NCBI) Gene Expression Omnibus (GEO). Thirty-seven PBMC samples from sepsis patients on acute phase of day 1 were divided into TIGIT-hi(n=19) and TIGIT-lo (n=18) groups for further comparison of the expression of IFN-γ. KEGG pathway enrichment analysis was conducted to identify differentially expressed genes (DEGs). **(E)** Transcriptional level of IFN-γ of sepsis patients with TIGIT-hi and TIGIT-lo expression. **p* <0.05, ***p* < 0.01; *ns*, not significant according to Student's t-test. **(F)** Top 12 enriched KEGG pathway of the DEGs involved in TIGIT-hi and TIGIT-lo sepsis patients.


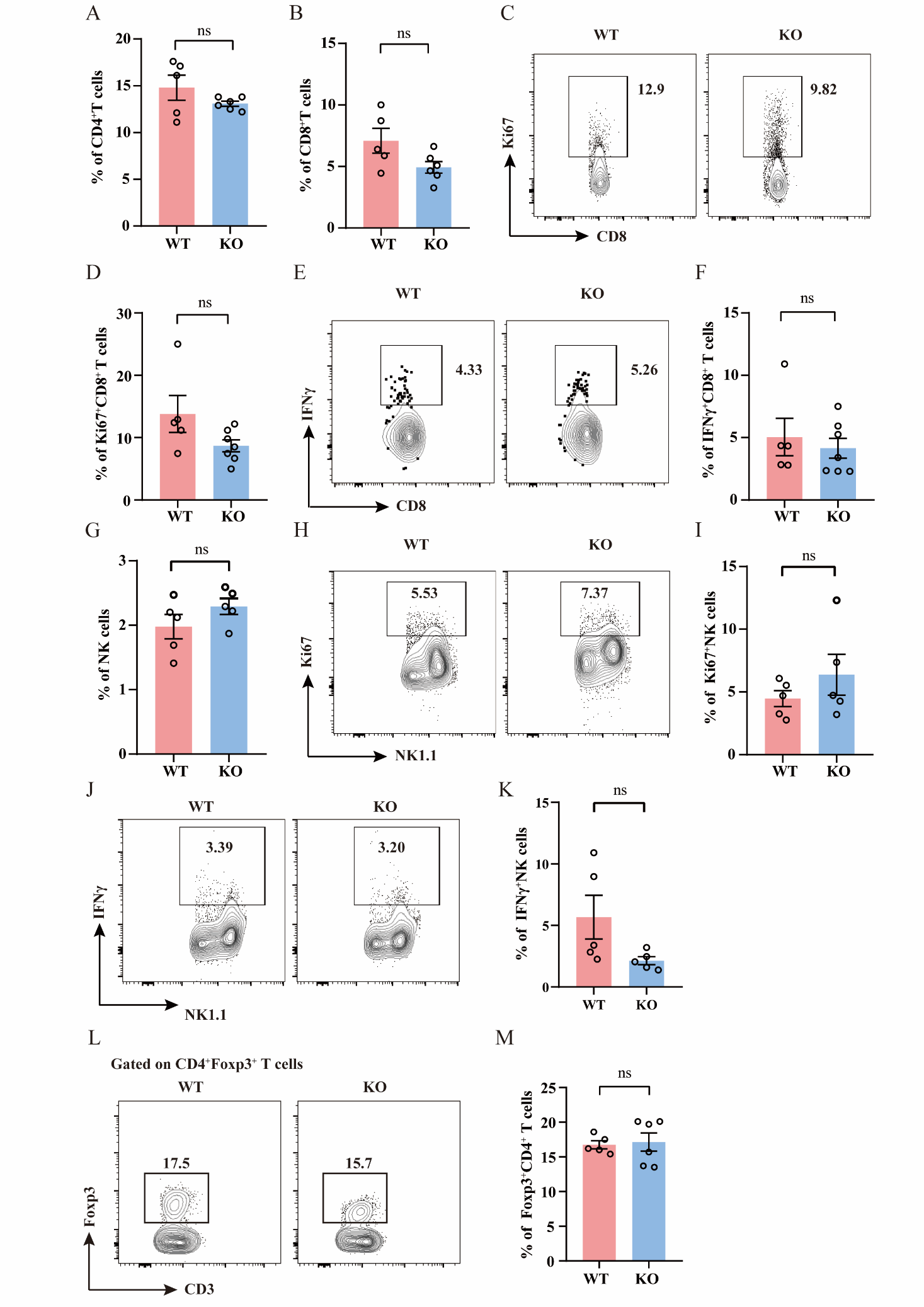


**Supplementary Figure 2. TIGIT deficiency showed no effects on Tregs, CD8^+^ T cells and NK cells during CLP-induced sepsis.** Statistical graphs showing the percentage of CD4^+^T cells **(A)** and CD8^+^T cells (**B)** from splenocytes of wildtype (WT) and TIGIT^-/-^ mice underwent CLP. **(C, D)** Representative flow cytometry counter plot and statistical graph of Ki67 expression in CD8^+^ T cells from splenocytes of WT and TIGIT^-/-^ septic mice. **(E, F)** Representative flow cytometry counter plot and statistical graph of IFN-γ expression in CD8^+^ T cells from splenocytes of TIGIT WT and TIGIT^-/-^ septic mice. **(G)** Statistical graph showing the percentage of NK cells from splenocytes of wildtype (WT) and TIGIT^-/-^ mice underwent CLP. **(H)** Representative flow cytometry counter plot of Ki67 expression in NK cells from splenocytes of TIGIT WT and TIGIT^-/-^ septic mice. **(I)** Statistical graph of Ki67 expression in NK cells from splenocytes of TIGIT WT and TIGIT^-/-^ septic mice. **(J)** Representative flow cytometry counter plot of IFN-γ expression in NK cells from splenocytes of TIGIT WT and TIGIT^-/-^ septic mice. **(K)** Statistical graph of IFN-γ expression in NK cells from splenocytes of TIGIT WT and TIGIT^-/-^ septic mice. **(L, M)** Representative FACS counter plots and statistical graphs showing the percentage of Foxp3^+^CD4^+^Treg cells from splenocytes of wildtype (WT) and TIGIT^-/-^ mice. Comparison between WT and KO were analyzed using Student’s *t*-test. **p* < 0.05, ***p* < 0.01; *ns*, not significant.

## Supplementary Tables

**Supplementary Table 1.** Quantitative PCR primers sequences.

| **Name** | **Sequences** |
| --- | --- |
| TIGIT  CD155 | Forward: GCACGATAGATACAAAGAG  Reverse: ACTGAAGACTGAAGCGACA  Forward: GAGGCAGTAGAAGCACCAATGC |
|  | Reverse: GGTGACCATTGGCAGAGATGCA |
